# Recent advances in understanding/managing eosinophilic esophagitis in adults

**DOI:** 10.12688/f1000research.6942.1

**Published:** 2015-08-20

**Authors:** David A. Katzka

**Affiliations:** 1 Division of Gastroenterology and Hepatology, Mayo Clinic, Rochester, MN, USA

**Keywords:** eosinophilic, esophagitis

## Abstract

It is an exciting time for research in eosinophilic esophagitis (EoE). As a new and increasingly prevalent disease, it is receiving considerable attention in the medical world, resulting in a flood of new insights. Clearly, a genetic predisposition seems likely with the identification of abnormalities in thymic stromal lymphopoietin (TSLP), calpain14, and eotaxin-3 genes. There are also well-defined abnormalities described in esophageal epithelial barrier function in these patients. The relationship between gastroesophageal reflux disease (GERD) and EoE remains unclear, but emerging data suggest that the concept of proton pump inhibitor responsive esophageal eosinophilia (PPIREE) may retain less importance, as this subset of patients becomes a likely subset of EoE in general. Finally, we approach the looming issue of long-term maintenance therapy. Although we lack adequate specific data on how to provide long-term pharmacologic treatment, studies clearly show that for most patients, this is a progressive disease that warrants such consideration.

## Introduction

It is not often in medicine that we have the excitement of researching and understanding a new disease, but this has occurred with eosinophilic esophagitis (EoE). Neither described nor understood until the 1990s, a wealth of information on EoE has emerged over the past two decades. It is remarkable that within such a relatively short period of time, major advances in understanding its molecular and clinical pathogenesis, disease characteristics, and treatment options have been defined. Nevertheless, a need for greater understanding of this disease, including basic questions with regard to diagnosis and treatment, remains. The goal of this review is to present many of the known highlights of EoE with identification of the key questions that need to be answered in the future.

## Pathophysiology

EoE appears to fall within the paradigm of an allergic disease with a genetic predisposition. In children, several genetic abnormalities have been identified, including in the eotaxin-3 gene and thymic stromal lymphopoietin (TSLP) ^1– 5^. More than just association, these abnormalities may have clear functional consequences. For example, the eotaxin-3 gene codes for eotaxin, which is a strong chemoattractant for eosinophils ^6^. TSLP, on the other hand, is a key promotor of T(H)2 responses found in EoE ^3^. There are also reported abnormalities in the epidermal differentiation complex (EDC) genes that code for filaggrin, an important protein regulator of the epithelial barrier ^7^ and the calpain 14 gene ^8^. These data are not surprising, as allergic disorders, such as asthma, atopic dermatitis, and seasonal allergies, commonly affect multiple family members and are thought to also have a genetic predisposition.

The proof that EoE is an allergy-based disease comes from several types of data. The first is in animal models in which EoE has been recapitulated in a mouse model ^9^. Interestingly, there appears to be a “two-hit” pathway in which animal priming with first airway disease (by administration of intratracheal Aspergillus) or skin disease (by subcutaneous injection with ovalbumin) is needed to induce esophageal eosinophilia ^10^. With this model, a careful analysis of the Th2 allergy pathway found in EoE has revealed multiple levels of involvement including an initial exposure of food antigens to dendritic cell recognition in the esophageal epithelium, epithelial secretion of key cytokines and eosinophil attractants, and recruitment of eosinophils and mast cells leading to esophageal inflammation and fibrosis ^11, 12^. Data in adults that corroborate these hypotheses include confirmation of the Th2 reaction histologically, identification of elevation of similar cytokines including eotaxin, and the frequent presence of extraesophageal allergies in patients ^13– 15^. This last point includes the finding that airway allergies commonly precede the onset of EoE. Some of the most compelling data suggesting that EoE is an allergy-based disease come from response to therapy. Specifically, medications such as steroids, which are useful in allergic diseases, are effective in inducing histologic remission in EoE ^16– 18^. Moreover, removal of food antigens by using an elemental diet (i.e. a diet devoid of food antigens) leads to histologic remission in close to 100% of EoE patients ^19– 22^.

Other important mechanisms have also been postulated as being contributory to EoE. One area of investigation includes how food antigens putatively gain access to the esophageal epithelium. Towards this end, investigators have demonstrated that patients with EoE have altered esophageal epithelial permeability, as shown through Ussing chambers containing biopsies and by an endoscopically placed mucosal impedance probe ^23^. Histologically, the epithelium in EoE demonstrates the presence of dilated intercellular spaces consistent with these physiologic findings. Furthermore, abnormalities in tight junction proteins, an important regulator of intercellular space diameter, have been shown in EoE tissue, some of which may become normal with the use of steroids ^6^. Thus, a clear pathway of how antigen penetration into esophageal epithelium, with subsequent recognition by the dendritic cell, is evolving.

In adults, an extremely important aspect of pathophysiology that is being explored is the mechanism of fibrosis in the esophageal wall with subsequent ring and stricture formation that commonly accompanies EoE. Investigators have clearly shown that in EoE, there is a secretion of fibrogenic peptides with esophageal remodeling ^24^ that may improve with steroids ^25^. The potential for this fibrosis is presumably related to ongoing uncontrolled esophageal inflammation. Furthermore, a recent fascinating study shows that IgG4, a known immunoglobulin associated with fibrosis in other diseases ^26^, may also be important in EoE.

Finally, there are compelling data that EoE is a genetic disease. Demographic data show that a personal and family history of allergies is common in patients with EoE ^14^. It has also been described in first-degree relatives both vertically and horizontally ^27, 28^. As previously noted, two genetic abnormalities have been identified in up to 50% of children.

## Diagnosis

As in an early description of any new disease, a classic set of characteristics emerges. In EoE, it became readily apparent that these characteristics include its occurrence in white male children and young adults, a presentation with dysphagia, and an endoscopic appearance of rings, furrows, white exudates and strictures associated with esophageal eosinophilia ^29– 32^. As recognition of EoE has evolved, it has become clear the these characteristics are not entirely specific, particularly in patients with gastroesophageal reflux disease (GERD) ^33^. Indeed, the greatest diagnostic challenge in EoE is distinguishing it from GERD. This task is formidable for several reasons. Firstly, as discussed, clinical features overlap. Secondly, GERD is a common disease and may co-exist with EoE. Thirdly, recent data suggest that GERD may potentiate EoE but cause the initial dilation of intercellular spaces and the subsequent increase in esophageal permeability ^23^. Fourthly, the results of ambulatory pH monitoring do not lead to accurate differentiation of these two diseases in all patients ^34^. Fifthly, histologically there are no obvious routine histologic markers nor special stains (e.g. of mast or eosinophil-derived proteins) that distinguish these two diseases ^35, 36^. Finally, proton pump inhibitors (PPIs) are therapeutic not only in GERD but also in EoE, where they may have cytokine-blocking effects independent of the acid-suppressing mechanism ^37, 38^. As a result, this has led to a newly defined category of patients, labeled PPI-responsive esophageal eosinophilia (PPIREE). Whether this truly represents a different phenotype of disease or a combination of EoE and GERD is unclear, although preliminary data suggest that PPIREE patients may eventually progress to EoE. It is the author’s opinion that categorization should be based on the terms EoE, GERD, and an overlap of the two ^39^.

Just as the typical characteristics of EoE are not diagnostic, we are starting to observe patients with atypical features of EoE. For example, whereas it was assumed that all adults with EoE have dysphagia, some patients are now being seen with heartburn or nausea as their presenting symptom ^40^. This is common in children where the isolated inflammatory response might be expected to produce such symptoms but was not expected in adults ^40^. Similarly, approximately 5% of adult patients will have a normal endoscopic appearance. These data must be considered carefully, however, as endoscopy is relatively insensitive to the detection of esophageal strictures when compared to barium esophagography ^18^. Thus, a normal endoscopic appearance may not reflect a truly normal esophagus.

With the lack of a gold standard for the diagnosis of EoE, establishing the diagnosis at this point still necessitates a combination of compatible clinical, endoscopic (and perhaps, radiologic), and histologic criteria, as recently defined ^30^. Needless to say, this definition will continue to evolve.

## Treatment

Before discussing specific treatment options, it is important to understand some of the fundamental questions on treatment that have not been answered. The most essential question is what is the desired endpoint of treatment? The present options are symptoms, histology or both. Although symptom resolution is a desirable endpoint of any therapy, it is problematic in EoE where symptom resolution correlates poorly to other important endpoints, such as histology ^18^. This is not surprising in adults, as short-term eradication of the esophageal epithelial inflammatory response does not lead to improvement of the fibrotic strictures that typically generate the symptoms of dysphagia. Another confounding issue in assessing symptoms is the lack of a standardized and comprehensive means of accurately measuring response. A study in press from the Swiss Eosinophilic Esophagitis Group will contribute greatly to resolving this problem, but, at this point, many physicians do not appreciate the subtle and adaptive behaviors that EoE patients adopt to cope with their disease and which can lead to inaccurate assessment of symptoms ^41^. The third issue is in the way we define treatment groups. For the past 5 years, patients with esophageal eosinophilia who respond to PPIs are termed as either GERD patients or PPI-responsive eosinophilia. As data continue to accumulate in this area, particularly histologic and cytokine data, it appears that those patients with PPIREE, that is patients with the phenotype of EoE who do respond to PPIs, are likely a subset of EoE and will be referred to in that manner.

The use of histology as a therapeutic endpoint also leads to several potential pitfalls. The first is in defining if the measurement of eosinophils is the most important (if not the only) correct histologic parameter to follow. Other pathologic features accompany esophageal eosinophilia, such as the elongation of rete pegs, basal zone hyperplasia, an increased number of mast cells, and dilation of intercellular spaces ^42^. It is unclear if all of these findings need to become normal in order to define a complete response. It also needs to be determined to what level eosinophils need to be reduced (or eliminated) to constitute a biologically meaningful response. Thus, in reading studies on EoE evaluating therapy, endpoints vary with different levels of eosinophil reduction including absolute numbers (e.g. <5) or percentage reduction. The main reason for this inability to establish a firm therapeutic histologic endpoint is the lack of knowledge of what is the natural history of EoE in the presence of these varied but sustained eosinophil levels. More specifically, we lack the knowledge of whether reduction to 15, 5 or 0 eosinophils per high power field prevents fibrosis and the progression of disease to strictures. Similarly, pathologists debate whether this measurement of eosinophils should be maximum or average per high power field and if all fields examined should be held to the same standard. With this in mind, the gold standard at this time remains elimination of all esophageal eosinophils, though, realistically, this is hard to achieve in all patients.

With these caveats in mind, medical treatment consists of pharmacologic and diet therapy. The first medication that should be considered in a patient with EoE is a PPI. Although one might define a response as indicating a patient with PPIREE, in a patient with classic features of EoE, I still consider this a first-line response. In patients with a typical phenotype of EoE, studies suggest a 30–60% initial response to PPI, but likely closer to 30% ^30, 43^.

From a theoretical point of view, diet therapy for EoE appears most logical. This logic stems from the knowledge that food antigens trigger the T(H)2 allergic response and therefore preventing exposure to these foods putatively eliminates the disease. Unfortunately, determining specific foods that initiate this response in individual patients is difficult, owing to the lack of reliable non-invasive tests to identify culpable food antigens. In a recent meta-analysis, antigen determination in EoE through routine allergy testing in adults was only 45% accurate ^44^. As a result, endoscopy followed by biopsy remains the only test at this time that reliably evaluates the effects of foods on esophageal inflammation. This can lead to up to ten endoscopies required for individual patients to find these foods using a series of additions and withdrawals of potential dietary constituents followed by esophageal biopsies ^45^. Although newer methods of esophageal mucosa sampling are being developed, they are not ready for general use at this time. On the other hand, a recent meta-analysis of diet therapy demonstrated that six-food elimination and elemental diets led to remission in 75 and 95% of patients ^44^. Although the six-food elimination diet has appeared unattractive to patients given the commonality of the foods eliminated, variations of this diet are gaining more popularity. For example, preliminary work is being performed for four- and two-food elimination diets ^46^. Even so, as the population embraces current trends of avoiding gluten and dairy (the two most common food antigens that trigger EoE), elimination diets are becoming more acceptable to patients.

Although not a pharmacologic therapy, endoscopic dilation is an important part of therapy in EoE, particularly in adults where strictures are common. Early literature that evaluated dilation in patients with EoE suggested a high rate of perforation, warning against its use or at least suggesting marked caution be taken ^47^. With the publication of far larger studies, a recent review suggests that the frequency of perforation is 1%, with most patients who sustain perforation responding to non-operative therapy ^48^. On the other hand, these studies demonstrate that dilation should be done gradually and carefully and perhaps over multiple sessions. With this in mind, dilation alone has been shown to be as effective in reducing symptoms of EoE as steroids in 2-year follow-up, although mechanical therapy directed at strictures does not improve histology ^49^. Rather than being viewed as an either/or therapy, in most adult patients therapeutic response is commonly achieved with a combination of medical and dilation therapy. This is particularly true in patients with marked esophageal narrowing, severe symptoms, or a history of food impaction.

One of the ongoing debates occurring in the field of EoE at present is the role of maintenance therapy. On the one hand, although EoE is not known to be associated with malignancy, there is concern that it leads to morbidity and chronic use of steroids. Furthermore, relapse is almost universal, there is a significant effect on quality of life, and severe complications such as food impaction or Boerhaave’s syndrome ^49, 50^ may occur. Recent data also suggest that untreated inflammation eventually leads to stricture formation in most patients ^50^. As a result, physicians are starting to define subsets of patients who might benefit from maintenance therapy. These include patients with EoE who respond to PPIs, patients effectively managed with diet therapy, and those who have a rapid relapse of symptoms and/or eosinophilia, severe stricturing disease such as in small caliber esophagus, or a history of food impactions or perforation. Whether all patients will ultimately require maintenance therapy is unclear.

If it is decided that chronic pharmacologic maintenance therapy is needed, unfortunately it is not clear what dose of medication to use. For PPIs, there are no randomized studies examining their role in maintenance, let alone whether once- or twice-daily dosing is effective. For topical steroids, there is only one randomized trial demonstrating that 0.5 mg of budesonide daily is suboptimally effective ^51^. At this point, anecdotal advice that varies from 1 mg every other day to 1 mg twice daily has been used to establish symptomatic and/or histologic remission.

## Future directions

There are numerous areas that need to be elucidated further in the field of EoE, but several are more pressing than others. The first is to find a “gold standard” for the diagnosis of EoE. As discussed, consensus guidelines dictate that the diagnosis is made with use of compatible clinical, endoscopic, and histologic characteristics. Genetic testing, as discussed previously, may fulfill this role, although positive testing is not found in all patients. The second area is in developing a test that is less expensive and invasive than endoscopy to assess histologic activity. This is important for monitoring therapy. This is particularly true in patients undergoing diet therapy where it can take up to ten endoscopies to determine the specific foods to which the patient is allergic. Candidate devices include the esophageal string test ^52^ and the cytosponge ^53^. Both are office-based procedures that do not require anesthesia. The hope for an easier test (such as through blood samples) is present, but results have not approached a level of accuracy amenable for practice. Finally, identification of subtypes of EoE, likely based on phenotype and/or genomic differences, will be helpful to define prognosis and predict the need for more intensive immediate and long-term therapy. Already, recent studies from the laboratories of Dr Marc Rothenberg (who has been pivotal in finding these genetic variations) have demonstrated an abnormal molecular profile in EoE patients which may also predict response to steroids ^54, 55^.

## Conclusion

EoE is a newly identified disease in which tremendous progress has been made in a matter of a couple of decades. From a classic description of an ostensibly rare disease, EoE is now a disease whose mechanism is well understood and has been well characterized, clinically, endoscopically, histologically, and genetically. Excellent and relatively safe treatment options are already available. Future clinical research on EoE is likely to concentrate mostly on the identification of a gold standard for diagnosis, the determination of factors that predict severe disease and response to treatment, and the role of maintenance therapy as we gain further understanding of the long-term complications of this disease.
