# Supplementary material for: Recent advances in understanding/managing eosinophilic esophagitis in adults
Source: F1000Res. 2015 Aug 20;4(F1000 Faculty Rev):592. [Version 1] doi: 10.12688/f1000research.6942.1 (PMC4544371; doi:10.12688/f1000research.6942.1)
Supplement: Peer review history

# Peer review: Referee response for version 1

**DOI:** 10.5256/f1000research.7475.r10034

**Published:** 2015-08-20

**Authors:** Jonathan Markowitz

**Affiliations:** 1 Pediatric Gastroenterology, Greenville Hospital System University Medical Center, Greenville, SC, USA

I have read this submission. I believe that I have an appropriate level of expertise to confirm that it is of an acceptable scientific standard.

# Peer review: Referee response for version 1

**DOI:** 10.5256/f1000research.7475.r10033

**Published:** 2015-08-20

**Authors:** Marc Rothenberg

**Affiliations:** 1 Cincinnati Children's Hospital Medical Center, Cincinnati OH, USA

No further comments provided.

I have read this submission. I believe that I have an appropriate level of expertise to confirm that it is of an acceptable scientific standard, however I have significant reservations, as outlined above.

# Peer review: Referee response for version 1

**DOI:** 10.5256/f1000research.7475.r10032

**Published:** 2015-08-20

**Authors:** Chris Liacouras

**Affiliations:** 1 Division of Gastroenterology, The Children's Hospital of Philadelphia, Philadelphia, PA, UK

I have read this submission. I believe that I have an appropriate level of expertise to confirm that it is of an acceptable scientific standard.
